# Clinical PathoScope: rapid alignment and filtration for accurate pathogen identification in clinical samples using unassembled sequencing data

**DOI:** 10.1186/1471-2105-15-262

**Published:** 2014-08-04

**Authors:** Allyson L Byrd, Joseph F Perez-Rogers, Solaiappan Manimaran, Eduardo Castro-Nallar, Ian Toma, Tim McCaffrey, Marc Siegel, Gary Benson, Keith A Crandall, William Evan Johnson

**Affiliations:** Department of Bioinformatics, Boston University, Boston, MA USA; Genetics and Molecular Biology Branch, National Human Genome Research Institute, National Institutes of Health, Bethesda, MD USA; Division of Computational Biomedicine, Boston University School of Medicine, Boston, MA USA; Computational Biology Institute, George Washington University, Ashburn, VA USA; Division of Genomic Medicine, George Washington University, Washington, DC USA; Division of Infectious Disease, George Washington University, Washington, DC USA; Department of Computer Science, Boston University, Boston, MA USA; Department of Biology, Boston University, Boston, MA USA

## Abstract

**Background:**

The use of sequencing technologies to investigate the microbiome of a sample can positively impact patient healthcare by providing therapeutic targets for personalized disease treatment. However, these samples contain genomic sequences from various sources that complicate the identification of pathogens.

**Results:**

Here we present Clinical PathoScope, a pipeline to rapidly and accurately remove host contamination, isolate microbial reads, and identify potential disease-causing pathogens. We have accomplished three essential tasks in the development of Clinical PathoScope. First, we developed an optimized framework for pathogen identification using a computational subtraction methodology in concordance with read trimming and ambiguous read reassignment. Second, we have demonstrated the ability of our approach to identify multiple pathogens in a single clinical sample, accurately identify pathogens at the subspecies level, and determine the nearest phylogenetic neighbor of novel or highly mutated pathogens using real clinical sequencing data. Finally, we have shown that Clinical PathoScope outperforms previously published pathogen identification methods with regard to computational speed, sensitivity, and specificity.

**Conclusions:**

Clinical PathoScope is the only pathogen identification method currently available that can identify multiple pathogens from mixed samples and distinguish between very closely related species and strains in samples with very few reads per pathogen. Furthermore, Clinical PathoScope does not rely on genome assembly and thus can more rapidly complete the analysis of a clinical sample when compared with current assembly-based methods. Clinical PathoScope is freely available at: http://sourceforge.net/projects/pathoscope/.

**Electronic supplementary material:**

The online version of this article (doi:10.1186/1471-2105-15-262) contains supplementary material, which is available to authorized users.

## Background

Despite recent advances in diagnostic and preventative medicine, infectious diseases still account for a large proportion of the disease burden and mortality worldwide, particularly in low-income areas and developing countries [1]. Current clinical diagnostic tests for identifying infection-causing pathogens utilize limited technologies such as polymerase chain reactions (PCR), Sanger sequencing, or cell culture. These methods typically focus on identifying only a single pathogen at a time and often lack the specificity required to distinguish between closely related species or strains of the same species. Bacterial cultures can accurately identify culturable pathogens, but usually require 4–5 days to complete and cannot be conducted for all pathogens [2]. Microarray technologies, such as the Virochip [3], have been shown to be useful in the space of pathogen identification. Microarrays, such as these, are designed to detect known pathogens through the use of high-sensitivity probes and isotype novel pathogens using probes that map to conserved genomic regions. While useful for broad spectrum screening of clinical samples, this technology is limited in that probes must be continually designed and updated to support the ever-growing number of genomic sequences in public databases.

In recent years, researchers have taken advantage of innovations in sequencing technologies to more rapidly identify and characterize pathogens responsible for disease outbreaks, including the West Nile Virus [4], H1N1 influenza [5–7], cholera [8], *Escherichia coli* [9–12], *Salmonella* [13], and antibiotic resistant *Klebsiella pneumoniae* [14]. Traditionally, sequencing a single sample has taken as long as several days or weeks using the most common platforms. Recent commercial efforts, however, have reduced this time to a few hours or days, projecting within the next few years sequencing runs of less than an hour with a cost of under one hundred dollars [15]. Once these technologies become widely accessible, the use of sequencing as a diagnostic tool in the clinic will have great potential for more personalized medical applications. The rapid and accurate analysis of next-generation sequencing data, however, remains a challenge for many reasons. The sheer volume of data, for example, is difficult to analyze without significant computational resources (e.g., a typical sequencing run on the Illumina HiSeq 2500 can yield 300 million reads requiring 30 GB of storage capacity and significant RAM requirements for processing) [16]. Furthermore, DNA from host genomes or commensal species will often dominate clinical samples and sequencing error can swamp out diagnostic signal. These challenges highlight the need for the development of highly sensitive algorithms that can distinguish among closely related pathogenic strains in a computationally efficient manner.

Current sequencing-based diagnostic methods [17–23] require thousands of reads from the pathogen and include computationally intensive steps such as genome assembly, multiple genome alignments, extensive homology searches, and/or phylogeny estimation, with some methods taking upwards of three days to complete a single run [17]. Additionally, these methods fail to accurately identify pathogens at the strain level and will often assign ambiguously aligned reads to higher taxonomic levels which may lead to a nonspecific or incorrect diagnosis and the administration of ineffective clinical treatments. Such was the case during the European outbreak of hemorrhagic *Escherichia coli*, which resulted in 3,800 infections and 54 deaths across 13 countries due to a 3-week delay in appropriate intervention [9]. The challenges encountered when diagnosing viral and bacterial pathogens in the clinic reinforce the need for a streamlined sequencing protocol and a highly sensitive computational method by which strain specific identification can be rapidly achieved. By helping clinicians to direct treatment and avoid misdiagnoses, the identification of viral and bacterial pathogens in clinical samples will directly benefit patients suffering from a variety of infectious diseases [24]. In particular, assigning a viral rather than bacterial cause to an infection may help alleviate the antibiotic overuse that is common in clinical practice today [25]. Recent editorials and reviews express concern that analysis, rather than data generation, is likely to be the limiting factor for sequence-based clinical pathology; thus, clearly highlighting the need for ‘clinic-ready’ software tools and approaches [2, 26–29].

Here we present Clinical PathoScope, a rapid alignment and filtration pipeline for accurate viral and bacterial pathogen identification using unassembled sequencing data. Using a variety of clinical samples and simulated scenarios, we demonstrate our method’s ability to differentiate between pathogens, identify multiple pathogens in a single clinical sample, and identify the closest relative to highly mutated and novel strains. Clinical PathoScope builds on the previous success of PathoScope v1.0 [30], which capitalizes on a Bayesian statistical framework to process an alignment file and provide posterior probability profiles of organisms present. While PathoScope v1.0 showed success when used with purified samples, it was necessary to develop a method to remove potential contaminating sequences from the host and commensal microbes for host-dominated clinical samples. Clinical PathoScope incorporates the original PathoScope algorithm into a novel pipeline that allows users to go directly from metagenomic sequencing reads to a list of organisms present in a sample in one easy step and in a clinically relevant timeframe. For convenience, we provide bacterial and viral databases curated from NCBI; however, custom databases can easily be incorporated as well. Taken together, these features make Clinical PathoScope the fastest and most accurate pipeline currently in the literature for identifying strain-specific pathogens in clinical samples without the need for genome assembly. Clinical PathoScope (version 1.0) is freely available at: http://sourceforge.net/projects/pathoscope/.

## Methods

In order to develop the Clinical PathoScope framework, we have accomplished the following essential tasks for pathogen identification in clinical samples: 1) selection of the most appropriate alignment algorithm and parameters for optimal performance on clinical samples, 2) evaluation of filtering approaches to efficiently remove reads from a clinical sample that originated from host, non-target, or non-pathogenic genomes, and 3) the evaluation and comparison of Clinical PathoScope with existing approaches using multiple real datasets [see Additional file 1 for Clinical PathoScope development workflow]. Details regarding the specific methods evaluated, pipeline modules, and results observed are given in the subsequent sections. Finally, we have implemented these results into a highly sensitive and efficient pipeline that is user-friendly and approachable by physicians and researchers without the requirement of advanced computational expertise.

### Clinical PathoScope pipeline development & evaluation

The Clinical PathoScope pipeline consists of three primary steps: 1) optimized read alignment, 2) host and non-target genome filtration, and 3) ambiguous read reassignment. We developed the optimized Clinical PathoScope algorithm using a set of simulated clinical samples (described below) and later validated our method and compared our results against existing approaches using multiple clinical datasets, some of which are original to this publication.

#### Reference genome library curation and processing

One of the most important steps for the accurate identification of benign and pathogenic genomes is to build a comprehensive genome library containing all species and strains likely to be present in the sample. This is a critical step as Clinical PathoScope can only identify organisms or their nearest neighbors if they are present in the library. In order to maximize the characterization of all reads within a given clinical sample, our method aligns reads against three broad categories of reference genomes. The human host library consisted of two sequences totaling 3.2 gigabase-pairs (Gbps); the GRCh37/hg19 build of the human genome, as well as the human ribosomal DNA sequence [GenBank:U13369]. The ribosomal reference was included in order to remove several false positive alignments to viral genomes that share sequence similarity with human ribosomal RNA (a list of these viral genomes is given in Additional file 2). The bacterial library was downloaded from NCBI ( ftp://ftp.ncbi.nlm.nih.gov/genomes/Bacteria/all.fna.tar.gz, 12/15/12) and contained 2,402 complete reference genomes and 1,759 plasmid sequences. In all, this bacterial library consisted of 7.7 Gbps of DNA sequence. Due to restrictions enforced by some of the aligners with regard to index size, it was necessary to split this library into two smaller segments to facilitate proper alignment. Finally, the viral library was also obtained from NCBI ( ftp://ftp.ncbi.nlm.nih.gov/genomes/Viruses/all.fna.tar.gz, 1/10/13). For genomes in which multiple segments were available, all segments for a given genome were concatenated into a single contiguous sequence with each segment separated by a series of null characters (N’s). In total, the viral library contained 3,738 complete genomes and 110 megabase-pairs (Mbps) of total sequence.

#### Generation of simulation study datasets

We simulated two sets of five *in silico* clinical samples to represent a variety of clinical scenarios including infections with two or more disease causing and benign pathogens, infections with a pathogen having closely related substrains (e.g. Human adenovirus), and infections with highly mutated pathogens. The first set of simulated samples was used to evaluate several alignment algorithms and to optimize the architecture of the Clinical PathoScope pipeline. The second set was then used to evaluate the efficacy of Clinical PathoScope alongside existing technologies. Importance was placed on implementing accurate mutation rates, genome diversity, and relative compositions. Functioning as positive controls, these data were essential to develop a robust pipeline for pathogen identification. Each sample was composed of human, bacterial, and viral sequences mimicking the microbiota found in sequencing data from nasopharyngeal samples during a respiratory tract infection [31, 32]. Specifically, 10 million 100-base reads were generated for each sample with 90% of reads originating from the host transcriptome (human RNA), 9% from bacterial genomes, and 1% from viral genomes. The first set of simulated samples contained sequencing reads from five bacterial and six viral genomes at various depths of coverage. This was essential to determine how each aligner and pipeline architecture performed with respect to the number of reads originating from each genome. The second set of simulated samples was designed as a more challenging and realistic dataset and was used to evaluate our optimized approach. Each sample contained sequences from six viral genomes and twenty-five bacterial genomes. The number of reads originating from each viral genome ranged from ten to 63,640. To determine a realistic bacterial landscape for these samples, we downloaded and aligned three anterior nares samples [SRA: SRS011105, SRS012291, SRS013637] from the Human Microbiome Project ( http://hmpdacc.org/HMASM/) and selected 25 of the most common bacterial strains (19 unique species) to be included in our simulation. The number of reads originating from each bacterial genome was determined by sampling a Gaussian distribution such that the number of bacterial reads per sample totaled 900,000. Reference genomes for each of the representative species were obtained from NCBI’s RefSeq database [33] and samples were simulated using the next-generation read simulator, Mason [34], employing its ‘Illumina sequencing’ error-model. Previously published species or kingdom specific mutation rates for SNPs and indels were applied to the human [35], bacterial [36], and viral [37] genomes to accurately capture the variability inherent in clinical samples. The simulated datasets are available for download on the PathoScope software distribution site and will be useful for benchmarking and comparing future metagenomic analysis pipelines. The specific parameters and code used to generate this dataset as well as accession numbers of reference genomes and actual read proportions of each genome within each sample are given in Additional file 3.

#### Alignment optimization

We evaluated and compared four publicly available alignment algorithms (Bowtie2.0.0 [38], BWA 0.6.2 [39], PBLAT 2.0.0 [40], SOAP2 2.21 [41]) based on three criteria, namely, 1) run time, 2) sensitivity and 3) specificity by aligning our first set of five simulated samples against the human, bacterial, and viral reference libraries described above [see Additional file 4 for aligner evaluation schematic]. Run time was measured as cpu minutes using 8 cores and a single 2.3 GHz AMD Opteron processor on the Boston University Medical Campus LinGA cluster. Using the resulting alignment files and the known origin of the reads, sensitivity was measured as the number of true positives divided by the number of true positives plus false negatives, and specificity was measured as the number of true negatives divided by the number of true negatives plus false positives. Our goal was to identify the algorithm and parameters that provided the best balance of our three evaluation criteria. Additionally, we examined the effect of varying the length of each read on the number of reads correctly aligned to the reference genomes using the first 25, 50, 75, and 100 base-pairs, as well as the full-length sequence. Evaluating variable read lengths served multiple purposes: 1) determining whether aligning the entire read was necessary, or if aligning a smaller segment of the read performed just as well, 2) identifying optimal sequence read size for future studies, and 3) evaluating whether aligning a smaller portion of the read can replace the need for a computationally intensive spliced-read alignment algorithm for reads from host/filter genomes that contain spliced gene transcripts. The version information, run commands, and alignment results for each algorithm and all parameters evaluated are included in Additional file 5 and Additional file 6.

#### Filtration optimization

We employed a computational subtraction methodology [42] in which reads are sequentially aligned against a series of reference genomes to determine their origin. For our purposes, we aligned reads against libraries of reference genomes originating from human, bacteria, or viruses. Within our pipeline, reads that align to the target library (e.g. viral library for virus detection) are retained while reads that align to the host (e.g. human library) and non-target (e.g. bacterial library) sequences are removed. The effects of varying the order of subtraction were examined by comparing the resulting alignment sensitivity, specificity, and pipeline run time using all six permutations of our three libraries. Additionally, we evaluated the effect of using the PathoScope expectation maximization (EM) algorithm [30] to minimize false positive mappings by reassigning reads with ambiguous alignments to their correct genome of origin. A detailed diagram of the overall experimental design is shown in Additional file 1. The subtraction methods evaluated for use in our pipeline as well as the optimal method are shown in Additional file 7.

### Clinical datasets

#### Prostate Cancer Cell Line (PCCL)

The PCCL dataset [43] has been leveraged in previous studies as a positive control and a means for comparing algorithm run time. This dataset is derived from a prostate cancer cell line infected with the human papillomavirus serotype 18. The RNA sequencing was performed using an Illumina GA II sequencer and 26,958,682 reads (40 bases each) were publically available [SRA:SRR073726].

#### New World Titi Monkey Adenovirus Outbreak (TMAdv)

Sequencing reads from two New World titi monkeys (*Callicebus cupreus*) infected with a highly divergent adenovirus [44] make up the second dataset used to evaluate Clinical PathoScope. The samples originated from an outbreak of an unknown virus in a colony of titi monkeys in California. Chen *et al*. obtained tissue samples from the lungs of two titi monkeys during necropsy and were sequenced together using the Illumina GA IIx for 73 cycles in both directions yielding 12,393,506 reads (73 bases). Chen *et al*. identified the cause to be a new highly divergent species of adenovirus that was subsequently assembled and so named Titi Monkey adenovirus (TMAdv). We supplemented our host library with the most closely related, fully sequenced simian species, *Callithrix jacchus* [GenBank:PRJNA46205]. As a positive control, we included the TMAdv genome in our target library and validated that Clinical PathoScope accurately distinguished the TMAdv from all other adenovirus genomes.

#### Tuberculosis in a Mummy

Sequencing reads from a 200 year old mummy infected with tuberculosis were obtained from a previous study [45] and used to evaluate Clinical PathoScope’s ability to detect bacterial pathogens. The sample was collected from lung tissue taken from the left side of the thorax of a mummified body. Pulmonary tuberculosis was suspected because of the cathectic state of the body and was confirmed by PCR analyses. As further validation, the sample was sequenced on the Illumina MiSeq instrument for 300 cycles in both directions yielding 5,541,400 reads with an average length of 297 basepairs; the reads were retrieved from Sequence Read Archive with accession number SRP018736. For analysis with Clinical PathoScope, the reads were split into 12,261,862 reads of approximately 100 bases in length.

#### 16S Amplimer Sequencing (16S)

In addition to testing our approach on *in silico* and previously published clinical datasets, we validated our approach on data from our own clinical samples. Under GW IRB-approved protocol #051140, unused deep endobronchial sputum samples acquired from three intubated subjects were obtained after the samples had been used for standard microbiologic testing and culture as directed by the medical team. A waiver of informed consent was used since the samples were being used as part of the standard of care for these subjects. Each subject was provided a handout detailing the study and given them the option to have their sputum samples excluded from the study. The bacteriological staining of aspirate samples revealed the presence of Gram-negative bacteria, and bacterial culture from aspirates identified abundant *Pseudomonas* (patients F1 and G1) and *Enterobacter* (patient H1), with opportunistic flora in all samples. All three patients were on an antibiotic treatment regimen prior to the collection of samples. Patient F1 was treated with a combination of aminoglycoside (gentamicin and tobramycin) and polymyxin (colistin) antibiotics; patient G1 was on gentamicin/tobramycin regimen only, and patient H1 was treated with third generation cephalosporin antibiotics (ceftazidime). In addition to clinical samples, we collected the bacterial DNA from gram-positive and gram-negative ATCC reference strains: *Staphylococcus aureus* (ATCC No. 25923 - MSSA), *Enterococcus faecalis* (ATCC No. 51299), *Pseudomonas aeruginosa* (ATCC No. 27853), *Escherichia coli* (ATCC No. 25922). Total DNA from these samples was isolated by centrifugation, and solubilization of the pellet using the Sigma GeneElute kit combined with a lysis buffer by mixing together the Gram + and Gram- buffers supplemented with lysozyme (2.115X10^6 units/mL), lysostaphin (200 units/mL), mutanolysin (5000 units/mL). Nanodrop and Qubit measurement of concentrations were used to quantify DNA. After DNA isolation, we amplified the 16S ribosomal RNA (rRNA) gene using the U1492R, Tm 49.44 (GGTTACCTTGTTACGACTT) and B27F, Tm 41.67 (AGAGTTTGATCCTGGCTCAG) universal primers using 800 ng of template. The amplimers were ligated into SMRTbells and sequenced on a Pacific Biosystems RS. The sequencing yielded an average of 4,127 reads per sample, averaging 1,178 bases long. For analysis with Clinical PathoScope, the PacBio reads from each sample were split into 100 base segments that were then treated as individual reads, generating on average 39,183 reads of 100 bases per sample. To accommodate the high identity of 16S RNA sequences from different bacterial species and strains, the alignment parameters for this dataset were tightened compared to the viral samples, allowing 1 mismatch per 100 bases during alignment, and allowing for multiple ‘best’ hits per read (e.g. Bowtie2 ‘k’ set at 1,000). These data were submitted to the NCBI Sequence Read Archive (SRA) database under accession number SRP028704.

### 16S phylogenetic inference

We took all genomes from GenBank’s RefSeq database belonging to *Pseudomonas*, *Enterobacter*, and *Acinetobacter* genera (56 taxa) and generated a BLAST database, which we queried with a full-length 16S rRNA gene sequence [46]. We selected one copy per species and aligned the resulting dataset using a secondary structure aware algorithm (Q-INS-i) as implemented in MAFFT [47]. We ran 10 independent Maximum Likelihood searches in RAxML [48] (1000 bootstraps) assuming a GTR nucleotide substitution model with gamma distributed rate heterogeneity. Additionally, we obtained diagnostic characters defining particular species using the phylogeny-aware algorithm implemented in CAOS [49].

### Clinical dataset preprocessing

The four clinical datasets were used to evaluate our Clinical PathoScope pipeline and to compare our method against previously published algorithms. A summary of these datasets is shown in Additional file 8. Extensive quality control was performed uniformly on each of the datasets to remove low quality and artificial sequences using PrinSeq [50] (-derep 123; -lc_method dust; -lc_threshold 40) and Cutadapt [51], respectively. For each read, bases having a Phred quality score less than 20 were trimmed from the 3′ end and reads with a median quality score below 20 were removed. Low complexity and redundant reads were determined using PrinSeq and removed along with adapter and primer sequences [see Additional file 9 for a complete list of adapter and primer sequences]. A minimum read length of 25 base pairs was strictly enforced for trimmed reads to facilitate accurate sequence alignment. Reads that failed to meet the length requirement were not considered for further analysis.

### Comparison to published algorithms

Clinical PathoScope was evaluated alongside two existing pathogen identification algorithms, RINS [19] and READSCAN [18] to emphasize the major differences in performance between assembly-based approaches and our implementation of computational subtraction with varying read length and ambiguous read reassignment. All three methods were compared based on their ability to rapidly identify the pathogens present in the clinical datasets described above. We also considered several published metagenomic-like pipelines such as CloVR-Metagenomics [52], IMSA [53], LMAT [54], and metAMOS [55] in the context of pathogen identification. These methods were of limited use in this context because of their significantly longer run times (see Results). Additionally, we tested MGmapper [56], KmerFinder [56, 57], and Tapir [58]. These approaches are webserver-based approaches, which in some cases have stand-alone downloadable software, however the stand-alone versions produced errors at implementation. Additionally, these methods were not designed for metagenomic samples and therefore have no mechanism for dealing with host sequences, and as a result these methods were not considered further in this study.

## Results and discussion

### Comparison of alignment algorithms

The internal parameters for each alignment algorithm were evaluated and tuned to maximize alignment sensitivity and specificity as well as to minimize run time by mapping reads from our first set of simulated samples to the reference libraries [see Additional file 5 and Additional file 6]. The average alignment results and confidence intervals of each algorithm using optimized parameters and read lengths are shown in Table 1. When aligning reads to the human library, SOAP2 was on average 30.5% faster than Bowtie2; however Bowtie2 had a 15.0% higher average sensitivity at 90.2% and a more consistent run time. For alignments to the viral library, PBLAT had the highest average sensitivity of 99.8%. Bowtie2 also achieved a high average sensitivity of 98.1% with an 80% reduction in average runtime compared with PBLAT. For alignments to the bacterial library, PBLAT had the highest average sensitivity of 98.9%; however, it took almost 20 times longer than Bowtie2, which had an average sensitivity of 79.8%. Overall, Bowtie2 offered the best combination of sensitivity, specificity, and speed when aligning reads against the human, bacterial, and viral libraries.

**Table 1:** **Simulation study alignment statistics using optimal model parameters**

	Human		Virus		Bacteria	
	Time (m)	Sensitivity	Time (m)	Sensitivity	Time (m)	Sensitivity
		Specificity		Specificity		Specificity
Bowtie2	8.2 ± 0.0	90.2 ± 0.0	3.3 ± 0.6	98 .1 ± 0.6	15.8 ± 1.6	79.8 ± 0.1
		100.0 ± 0.0		99.8 ± 0.2		100.0 ± 0.0
BWA	22.8 ± 3.2	89.9 ± 0.0	6.5 ± 1.4	76.8 ± 5.4	-	-
		100.0 ± 0.0		99.8 ± 0.2		-
SOAP2	5.7 ± 1.6	76.7 ± 0.0	3.9 ± 0.8	50.3 ± 5.4	23.3 ± 2.2	27.7 ± 0.0
		100.0 ± 0.0		99.9 ± 0.1		100 ± 0.0
PBLAT	61.2 ± 6.8	78.2 ± 0.0	16.7 ± 1.3	99.8 ± 0.1	306.3 ± 23.3	98.9 ± 0.0
		100.0 ± 0.0		99.6 ± 0.2		52.7 ± 0.0

Each aligner was used to align the first set of five simulated sequencing samples (10 million 100 base-pair reads) against each of the three genome libraries using optimal parameters. The average run time, sensitivity, and specificity as well as confidence intervals for each alignment are reported. BWA failed to run to completion with the bacterial library.

### Impacts of read length

We evaluated the effect of varying the length of each read used during alignment to further maximize the sensitivity, specificity, and minimize run time. Temporary read splitting and trimming allows clinical samples from any sequencing technology to be analyzed without compromising the speed and accuracy of the short read aligner or losing the alignment specificity of longer reads. For the five simulated samples, varying read length had a larger impact on runtime and sensitivity than adjusting internal parameters. Using Bowtie2 as our primary aligner, 10 million 50 base reads were aligned against the human library in an average 28 minutes, while aligning 100 base reads took on average 40 minutes. Depending on the reference library used, increasing read length may or may not increase sensitivity. Bowtie2 aligned 50 base reads to the human library with an average sensitivity of 90% and 100 base reads with a decreased average sensitivity of 75%. This trend can be explained by the splice junctions found in human transcriptome sequences. With fewer bases, the odds of a read spanning a splice junction are smaller and the read will be more likely to align. Conversely, when aligning reads against the bacterial and viral libraries, the average sensitivity is 10-20% higher using 100 base reads compared to 50 base reads [see Additional file 6 for complete results]. To evaluate if longer reads continue to increase sensitivity, a subset of 150 base simulated bacterial reads were tested. Results indicate that splitting the 150 base reads into 100 base and 50 base segments increased sensitivity by approximately 4 percent compared to leaving the reads at the full length of 150 bases. Thus, upon initiation, Clinical PathoScope splits all long reads into fragments with a maximum length of 100 bases.

### Library alignment and filtering order

Various filtration methods were evaluated in an effort to minimize computation burden and maximize accuracy. Five subtraction frameworks were evaluated: A) Naïve Approach, B) Target Centric, C) Target Centric + Reassignment, D) Host Centric + Reassignment, and E) Host Centric [Additional file 7]. In the target centric approaches, reads are first aligned against the target library followed by the host and non-target libraries. Conversely, in the host centric approaches, reads are first aligned against the host and non-target libraries and then against the target library. The naïve approach, or only aligning to the target library, took the least amount of time, but resulted in the highest number of false positives. While both the target centric and host centric filtration approaches yielded similar results in terms of accuracy, the target centric approaches required ten fewer minutes (~70% less total time) to run to completion than the host centric approaches. The target centric approaches were more efficient because a greater number of sequences were removed by initially mapping reads to the target library than to the host library, thus reducing computational burden for subsequent alignments. To determine the impact of the read reassignment algorithm, we compared the sensitivity of both target centric approaches by analyzing our second set of simulated samples. With viral pathogens as the target library, the target centric approach with read reassignment achieved an average sensitivity of 97.8% for species and strain level identifications. Without the reassignment algorithm, the target centric approach achieved an average sensitivity of 90.3% and 78.1% at the species and strain level, respectively. Concurrently, with bacterial pathogens as the target library, the target centric method with reassignment achieved an average sensitivity of 77.6% and 72.8% at the species and strain levels, respectively, compared with 52.8% and 41.7% for species and strain specific identifications without read reassignment. These dramatic improvements in sensitivity between methods with and without read reassignment demonstrate the necessity of this algorithm within the Clinical PathoScope pipeline. The performance difference between viral and bacterial identification can be directly attributed to the mixture of bacterial pathogens present in these simulated samples. When two very closely related strains of the same species are present in a given sample, Clinical PathoScope will tend to reassign reads which aligned to both strains to the strain with more uniquely identifying sequences. Details regarding identification accuracy of Clinical PathoScope with respect to each individual strain can be found in Additional file 3.

### Optimal Clinical PathoScope pipeline

**Figure 1:**
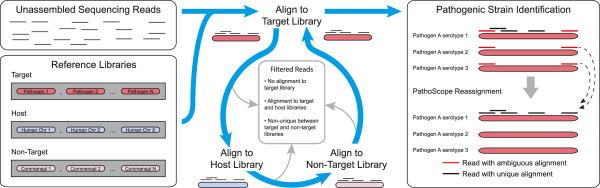
**Clinical PathoScope pipeline.** A computational subtraction method using varying sequence read lengths and ambiguous read reassignment. Unassembled sequencing reads are aligned against a target library containing reference sequences of the intended target(s) of identification (e.g. viruses). Reads aligned to the target library are then aligned to a host library. Any reads aligned to the host sequences are removed from further analysis. Next, reads are aligned against a library of known non-target sequences. Unaligned reads are then mapped back to the target library, allowing up to *k* alignments per read (e.g. k = 10). These alignments are subsequently passed to an expectation maximization algorithm in which ambiguous alignments are reassigned to their most probable genome of origin. Upon reassignment, a report detailing the pathogens identified and their relative abundances is produced.

The optimized Clinical PathoScope pipeline uses three reference genome libraries, four alignment modules and the original PathoScope read reassignment algorithm to identify pathogens in a given sample (Figure 1). First, all reads from a sample are mapped against the reference genomes of the organisms of interest (*target library*, e.g. viruses) using up to the first 100 bases of each read. This initial alignment results in the removal of the greatest number of sequences by eliminating reads without strong sequence similarity to the target genomes. Second, reads that aligned to the target library are aligned against the reference library of the host species (*host library*) using the first 50 bases of each read. This step allows for any residual host contamination to be identified and removed from the set of candidate reads originating from the target genomes. Third, reads which did not align to the host library are aligned against additional reference genomes (*non-target library*) known to be negative targets of the analysis and which may overlap with the candidate read set. Similar to step one, reads are aligned using the first 100 bases of each read to maintain high specificity. Reads which did not align to the non-target library are realigned to the target library allowing up to *k* alignments (e.g., we recommend *k =* 10 for viral detection) per read and subsequently passed to the read reassignment module in which reads with ambiguous alignments are reassigned to their putative correct genome of origin. In summary, any sequencing read contributing to the identification of a pathogenic genome must 1) align to the target genome library, 2) remain unaligned to the host genome library, 3) remain unaligned to the non-target library, and 4) retain its alignment to the target library. Finally, the pipeline produces a report detailing the number and proportion of reads originating from each genome identified in a given sample.

### Software implementation and distribution

The Clinical PathoScope pipeline has been implemented in open-source Python, and is freely available for download at: http://sourceforge.net/projects/pathoscope/. The software requires the user to supply a fastq read file (after conducting quality control), any number of target, host, and non-target library Bowtie2 indices. Furthermore, the user has the option of changing the pipeline alignment parameters using inputs in the configuration file. For convenience, our viral, bacterial, and human alignment indices are freely available for download on the software distribution website. Clinical PathoScope will output two alignment files in SAM format, one directly from the Bowtie2 alignment, and another after read reassignment. Finally, the pipeline will output a tab-delimited summary report containing the genomes found in the sample as well as read numbers and proportions assigned to each genome.

### Evaluation of clinical PathoScope on clinical data

Four clinical datasets were utilized to evaluate the efficacy of Clinical PathoScope across a variety of scenarios [see Additional file 8 for summary of datasets]. In addition, Clinical PathoScope was evaluated side by side with two previously published pathogen identification methods, RINS and READSCAN, on the basis of computational speed and accuracy at identifying pathogens in clinical sequencing samples.

#### Prostate Cancer Cell Line (PCCL)

Clinical PathoScope was able to rapidly decode the viral composition of this dataset; identifying the Human papillomavirus type 18 in fewer than 10 minutes. RINS and READSCAN both produced similar results; however, they required approximately four times the computational time to identify the pathogen, with run times of 89 minutes and 53 minutes, respectively (Table 2).

**Table 2:** **Run time comparisons of Clinical PathoScope and existing technologies**

		Average Run Time (minutes)		
Dataset	Target	Clinical PathoScope	RINS	READSCAN
Simulation	Virus	4.5	84.1	193.58
Simulation	Bacteria	13.1	1108.2	
PCCL	Virus	6.0	89.1	52.8
TMAdv	Virus	4.4	144.0	78.6
Mummy	Bacteria	25.0	1099	882

#### New World Titi Monkey Adenovirus Outbreak (TMAdv)

**Figure 2:**
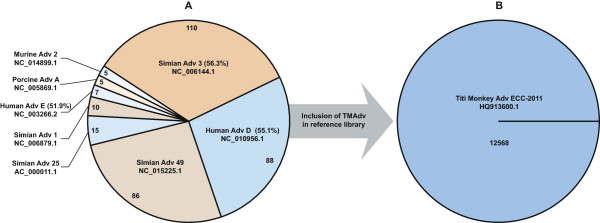
**Alignment variations with and without TMAdv in the target library. A)** Without the TMAdv present in the target library, Clinical PathoScope assigned reads to several adenovirus genomes. The identified genomes are displayed according to the proportion of total reads aligned to all adenovirus genomes. The pairwise nucleotide identities of several adenovirus subtypes to the TMAdv genome according to Chen *et al.* are given in parentheses. The Simian adenovirus 3 had the most reads aligned of all adenoviral genomes, which is consistent with its sequence similarity to the TMAdv. Additionally, the Human adenovirus D aligned the most reads of all human adenoviruses, which is consistent with the analysis of Chen *et al.* **B)** Inclusion of the Titi Monkey Adenovirus (TMAdv) in the target library resulted in the assignment of 12,568 reads to the TMAdv reference genome.

We examined Clinical PathoScope’s performance in two clinical scenarios using the TMAdv dataset. First, to evaluate our pipeline in cases where the exact strain is missing from the target library, we excluded the TMAdv strain from the target library. In this scenario, Clinical PathoScope assigned reads to several adenovirus species (Figure 2A). According to Chen *et al.*, the Simian adenovirus 3, which was the top ranked virus in the Clinical PathoScope result, is the closest phylogenetic relative to the TMAdv, with approximately 56% sequence similarity. Despite its highly divergent nature, Clinical PathoScope was able to successfully identify the closest phylogenetic neighbor of this novel species. Next, as a positive control, we included the TMAdv genome in our target library and validated that Clinical PathoScope accurately distinguished the TMAdv from all other adenovirus genomes (Figure 2B), identifying 12,568 reads from TMAdv. In their original analysis, Chen *et al.* used BLASTn [46] to identify 16,524 reads from TMAdv. This discrepancy can be explained by the fact that BLASTn is a much more sensitive algorithm than Bowtie2. This moderate increase in sensitivity, however, results in a dramatic increase in run time, with BLASTn requiring ten times longer to complete the alignment than Bowtie2 when TMAdv is the only sequence in the database. Therefore, with rapid pathogen detection as the goal, a Bowtie2-based approach clearly provides a reasonable trade-off between speed and sensitivity, whereas if genome assembly is the goal, a BLAST-based approach might be preferable (at the cost of computational efficiency). Despite aligning approximately 4,000 fewer reads than the analysis in the original publication, we were still able to obtain 22.0x coverage of the TMAdv genome. While it is clear that Clinical PathoScope aligned substantially more reads with the TMAdv genome in the target library than in its absence, we were still capable of generating a list of candidate relatives with read counts proportional to their sequence similarity with the TMAdv. Furthermore, Clinical PathoScope completed analysis of this dataset in less than 5 minutes (Table 2).

With the TMAdv genome in the reference library, both RINS and READSCAN were able to accurately identify the correct viral genome in the sample. When the TMAdv was removed from the library, RINS generated a single contiguous sequence consisting of only 156 reads which mapped to 6 different adenovirus genomes, none of which included the nearest phylogenetic neighbor. This shows that, while assembly may be possible in a given sample, the ambiguous mapping of a contig to multiple genomes provides little information pertaining to the true subspecies of origin. Additionally, RINS required 144 minutes to complete its analysis of this dataset. READSCAN assembled several contigs of varying lengths and read counts from 16–60 reads per contig. However, the adenovirus strains identified and ranked by READSCAN based on their relative genome abundance score [18] were inconsistent with phylogenetic relationships found by Clinical PathoScope and the original study [44]. Finally, READSCAN required approximately 80 minutes to analyze this dataset.

#### Tuberculosis in a Mummy

To demonstrate the performance of Clinical PathoScope with respect to bacterial pathogen identification, we analyzed a sample isolated from a mummy infected with tuberculosis. Using assembled contigs and comparative genomics, Chan *et al*. found evidence the deceased was infected with two *Mycobacterium tuberculosis* genotypes. Using patterns of deletions and SNPs, they concluded that both strains most closely resemble strain 7199/99, but also share similarities with strain H37Rv. When strain 7199/99 was included in the target database, Clinical PathoScope associates 32% of the reads with strain 7199/99 and 25% of reads with H37Rv. The majority of the remaining reads were split between additional *M. tuberculosis* strains and *Nocardia* species. Chan *et al*. also identified *Nocardia* species using their assembly approach. Clinical PathoScope successfully identified the most closely related strains and furthermore, only required 25 minutes to complete the analysis. While these results are in agreement with the author’s nearest-neighbor findings, we note that the number of novel *M. tuberculosis* strains in the sample (two unique strains according to Chan *et al.*) cannot be inferred from the Clinical PathoScope output alone. To successfully conclude the presence of two unique, novel strains in the sample, a more complex, assembly based approach is required. Neither RINS nor READSCAN performed well on this dataset, requiring 1099.0 and 882.25 minutes, respectively, to complete the analysis, likely due to the large average read size of 297 bases and the complexity of the bacterial database. RINS assembled 20,483 unique contigs of varying length and reported 1,044,193 unique alignments of these contigs to 2,293 bacterial genomes. While vast, these results are uninformative as to the specific strains present within the clinical sample. Several contigs were assigned to various *M. tuberculosis* strains in the RINS report; however, there was a tremendous lack of specificity with regard to the specific strains present in the sample. With thousands of other bacterial genomes identified and no metric for quantifying sequence abundance, the user is forced to interpret the results of thousands of contigs and millions of potential alignments, many of which are redundant or uninformative. READSCAN required less time to complete its analysis of the mummy dataset than RINS; however it also failed to generate a report detailing any of the identified pathogens. In their original publication, the authors demonstrate READSCAN primarily in the context of viral pathogen identification and note its performance improvements over previous methods. As can be observed from its run time on the mummy dataset, however, READSCAN has trouble scaling to larger bacterial datasets with many closely related strains of the same species.

#### *Bacterial species identification from 16S Amplimer Sequencing*(*16S*)

Clinical PathoScope was also tested on eight 16S amplimer samples (Accession: SRP028704), five originating from ATCC bacterial species, and three from patient tissue extracted from intensive care patients with suspicion of bacterial infections. As shown in Table 3, Clinical PathoScope was able to successfully identify the unique bacterial species in each of the first four ATCC samples with high accuracy. Furthermore, Clinical PathoScope was able to accurately identify the correct mixture of ATCC species in the fifth sample, assigning 30.4%, 30.2%, 21.2%, and 15.9% of the reads to *Escherichia coli*, *Enterococcus faecalis*, *Pseudomonas aeruginosa*, and *Staphylococcus aureus*, respectively.

**Table 3:** **Clinical PathoScope performance on the 16S amplimer dataset**

		Clinical PathoScope Results	
Accession	Sample type	Species identified	Reads assigned (%)
SRR949994	*S. aureus* ATCC No. 25923 MSSA	*S. aureus*	3,479 (98.0)
		*P. aeruginosa*	36 (1.0)
SRR949995	*E. faecalis* ATCC No. 51299	*E. faecalis*	2,351 (89.8)
		*S. aureus*	139 (5.3)
		*E. hirae*	44 (1.7)
		*P. aeruginosa*	42 (1.6)
SRR949996	*P. aeruginosa* ATCC No. 27853	*P. aeruginosa*	5,661(82.3)
		*E. coli*	1,021 (14.9)
SRR949997	*E. coli* ATCC No. 25922	*E. coli*	4,169 (94.7)
		*S. enterica*	66 (1.6)
SRR949998	Mixture of *E. coli, E. faecalis, P. aeruginosa, S. aureus* (above)	*E. coli*	14,280 (31.9)
		*E. faecalis*	14,306 (31.9)
		*P. aeruginosa*	8,771 (19.6)
		*S. aureus*	6,594 (14.8)
SRR950015	Clinical Sample (F1)	*A. baumannii*	4,889 (59.4)
		*P. aeruginosa*	3,177 (38.7)
SRR950024	Clinical Sample (G1)	*P. aeruginosa*	1,131 (94.5)
		*E. coli*	45 (3.8)
SRR950025	Clinical Sample (H1)	*E. aerogenes*	587 (85.9)
		*P. aeruginosa*	18 (2.6)
		*Erwinia sp. Ejp617*	19 (2.8)
		*E. coli*	18 (2.6)
		*S. enterica*	9 (1.3)
		*E. asburiae*	10 (1.5)
		*S. intermedius*	8 (1.2)

For the three patient samples, we observed that the first sample (F1) contained a mixture of *Acinetobacter baumannii* (57.6%) and *Pseudomonas aeruginosa* (40.4%), and that the other two samples (G1 and H1) were dominated by *Pseudomonas aeruginosa* (94.6%) and *Enterobacter aerogenes* (84.2%), respectively. To validate these results, we constructed a phylogenetic tree of 16S genes from all genomes in the reference library that reside within the three genera identified in the clinical samples [see Additional file 10]. We then visually inspected the read coverage pileup plots of 16S genes unique between identified species and their positions relative to phylogenetic neighbors [see Additional file 11]. We observed that read coverage is uniform across the genomes identified by Clinical PathoScope in each sample, resulting from the fact that they share 100% sequence similarity of their 16S genes. In contrast, we noticed large coverage gaps in the nearest phylogenetic neighbors, indicating that there were sequence variants in these regions that prohibited reads from aligning to these specific locations. This analysis further demonstrates the highly specific and accurate framework employed by Clinical PathoScope and its utility not only for strain-specific pathogen identifications, but also for 16S bacterial classification.

### Comparison to metagenomic pipelines

Clinical PathoScope has been designed to facilitate a rapid and streamlined approach to identify strain-specific pathogens in noisy clinical sequencing samples. We compared our method directly with two previously published algorithms, RINS and READSCAN, which were designed specifically for pathogen identification in clinical samples. Additional methods, such as PathSeq [17] and IMSA [53], were also considered. These methods rely on several BLAT and BLAST alignments in order to filter sequencing reads which can take several hours to days to complete depending on the number of reads in a given sample. To evaluate these types of approaches, we implemented a similar BLAST-based workflow and applied this workflow to our second set of simulated samples with the bacterial library as the target. This approach resulted in a substantial decrease in performance with only 48.3% and 34.8% sensitivity for species and strain-specific identifications, respectively. This BLAST-based approach required 55 hours and 26 minutes, which is 300 times slower than Clinical PathoScope. Therefore, these algorithms are not practical methods for rapid clinical diagnostics.

We further expanded our comparisons to metagenomic pipelines that were not specifically designed for the identification of pathogens in clinical samples but whose methods or modules may be useful for the task. We first considered the CloVR-Metagenomics pipeline which clusters raw sequencing reads to reduce redundancy followed by a simultaneous BLASTX and BLASTN analysis against RefSeq and COG in order to annotate each sequencing read. CLoVR-Metagenomics does not address the issue of host contamination and thus wastes computational time clustering and annotating sequences originating from the host which can account for >90% of the clinical sample. While very sensitive, BLASTN is notoriously slow and does not scale well to large metagenomic samples [54], making CLoVR-Metagenomics impractical for rapid strain identification. Furthermore, the redundancy reduction procedures employed by CLoVR-Metagenomics collapse sequences with 99% nucleotide similarity which could potentially remove reads that distinguish two closely related strains of the same species.

We also considered assembly-based metAMOS [55] and phylogeny-based LMAT [54]. metAMOS offers a rich suite of assembly algorithms and pathogen annotation methods, however it does not incorporate any methods to remove host or contaminating sequences. As a result, the assembly of sequencing reads from a host-dominated clinical sample would require an attempt to assemble the entire host genome. This will result in a substantial and unnecessary increase in computational time and these contaminating reads could result in high instances of false positive mappings. LMAT, a software package designed for taxonomy classification, claims accuracy only to the species level and does not report genome abundance information and thus cannot replicate the detailed pathogen report produced by Clinical PathoScope.

## Conclusions

Sequence-based diagnostic tools have the potential to revolutionize the treatment of patients in the clinic, particularly those suffering from viral and bacterial infections. As the run times and error rates of modern sequencing technologies rapidly decline, it is essential that software be developed to analyze these data in a manner that is both fast and highly sensitive in order to provide physicians with the most accurate information possible. We have implemented a novel pipeline for pathogen identification that overcomes many of the challenges faced by current sequence-based methods including clinically appropriate run time and subspecies specific assignment of sequencing reads. We have also demonstrated our method’s ability to identify multiple pathogens in a single clinical sample or the nearest phylogenetic neighbor of highly mutated or divergent species. Furthermore, Clinical PathoScope remained robust when analyzing datasets with lower than 1x coverage of the target genomes. It should be noted, however, that as coverage drops below 1x, the probability of sequencing a strain-specific segment of the target genome decreases. If these uniquely identifying reads are not sequenced and thus not present in the sample, Clinical PathoScope will tend to report the strain with the most aligned reads. Given that strain-specific reads do exist within a given sample, we expect the lower limit of coverage required to make a strain-specific identification to be comparable to our previously published results [30] in which we demonstrated the efficacy of our read reassignment algorithm with as low at 20% coverage of the genome.

The reference genome libraries used in this analysis contain all sequenced and assembled viral and bacterial genomes from NCBI’s RefSeq database. By avoiding genome assembly in favor of more rapid computation, Clinical PathoScope is limited in that it can only identify pathogens that are present in these reference libraries. While the libraries used in this study characterize the majority of known pathogens, they do not contain draft genomes. To broaden and extend the application of Clinical PathoScope in future studies, we allow the user to exchange, modify, or extend these libraries as more data becomes available.

By comparison with existing methods, we have demonstrated that our method is the fastest strain-level pathogen identification algorithm currently available in the literature. As the number of sequenced pathogens grows, the breadth of the reference libraries used with Clinical PathoScope will increase, thus expanding the search space required to assign sequencing reads to a specific genome of origin. While this increase in search space will result in a linear increase in run time, we assert that our method will not lose its computational advantage over existing methods.

In addition to faster run times and more accurate results, Clinical PathoScope offers a user-friendly implementation. With only two dependencies, Bowtie2 and the PathoScope reassignment algorithm, Clinical PathoScope can easily be installed and run on a standard desktop computer, facilitating a simplified workflow for the accurate identification of pathogens in clinical sequencing samples. While designed for use by computational biologists and biologists, the reports produced by Clinical PathoScope may prove useful to physicians as they provide a complete picture of the microbial community of a given clinical sample which may influence clinical diagnoses and treatment options.

### Availability and requirements

Project name: Clinical PathoScope.

Project home page: http://sourceforge.net/projects/pathoscope/.

Operating system(s): Platform independent.

Programming language: Python 2.7 or higher.

Other requirements: Bowtie 2.0 or higher.

License: GNU GPL.

Any restrictions to use by non-academics: License needed.

### Availability of supporting data

*Genome Reference Libraries*http://www.bu.edu/jlab/wp-assets/databases.tar.gz*.*

*Simulated read datasets:*http://sourceforge.net/projects/pathoscope/files/simulated_sample.fastq.gz/download.

*Prostate Cancer Cell Line (PCCL):* SRR073726; http://www.ncbi.nlm.nih.gov/sra/?term=SRR073726.

*New World Titi Monkey Adenovirus Outbreak (TMAdv):* SRA031285; http://www.ncbi.nlm.nih.gov/sra/?term=SRA031285.

*Tuberculosis in a Mummy:* SRP018736; http://www.ncbi.nlm.nih.gov/sra/?term=SRP018736.

*16S Bacterial Amplimer Sequencing* (*16S*): SRP028704; http://www.ncbi.nlm.nih.gov/sra/?term=SRP028704.

## Electronic supplementary material

Additional file 1: **Workflow employed to develop the Clinical PathoScope pipeline.** Three reference genome libraries were downloaded from NCBI. Four alignment algorithms were tested and evaluated on five simulated clinical sequencing samples. Each aligner was parameter tuned and optimized and Bowtie2 was selected as the choice aligner for the Clinical PathoScope pipeline. The order with which reads are aligned to the reference libraries was determined and the performance of Clinical PathoScope was evaluated using four clinical datasets. Furthermore, we compared our results against those produced by existing technologies. (PDF 19 KB)

Additional file 2: **Viral genomes with human ribosomal RNA contamination.** GenBank accession numbers and names of viral genomes showing sequence similarity to human rRNA sequences. (TXT 181 bytes)

Additional file 3: **Simulated data summary & code.** Genome accession numbers, read counts, mutation rates, and run commands used to generate the simulated sequencing samples. (XLSX 38 KB)

Additional file 4: **Alignment optimization variables and methods.** The internal parameters for each of the four aligners were varied and tuned. Additionally, the length of each read aligned was varied. For each unique aligner-parameter-read length configuration, the sensitivity, specificity, and run time when aligning the simulated samples against the reference genome libraries was calculated. (PDF 76 KB)

Additional file 5: **Commands and versions of alignment algorithms evaluated.** (DOCX 23 KB)

Additional file 6: **Results of all alignment runs.** (XLSX 41 KB)

Additional file 7: **Subtraction and filtration optimization methods.** Various filtration methods were tested in an effort to minimize computational burden and maximize accuracy. Approaches tested include A) Naïve Approach, B) Target Centric, C) Target Centric + Reassignment, D) Host Centric + Reassignment, and E) Host Centric. Post filtration, all reads are aligned against the target genome library. The resulting read alignments are reassigned to the correct genome of origin using the PathoScope Expectation Maximization algorithm. (PDF 39 KB)

Additional file 8: **Overview of clinical datasets used to evaluate Clinical PathoScope.** (XLSX 10 KB)

Additional file 9: **List of candidate primers and adapters used for quality control filtering.** (TXT 349 bytes)

Additional file 10: **Phylogeny of 16S genes for genera found in clinical samples.** We constructed a phylogenetic tree of 16S genes from all species in the reference library from the genera identified in the patient samples from the clinic. This tree was used to identify the nearest 16 s neighbor of the Clinical PathoScope diagnosis, and to check initial mapping read coverage of 16 s genes. (PDF 177 KB)

Additional file 11: **Read coverage for 16S genes and nearest phylogenetic neighbors.** A) F1, B) G1, and C) H1 16S clinical samples (top frame: overall coverage, bottom frame: ‘pileup’ plot for a selected sets of the reads). Coverage for the ‘nearest’ phylogenetic neighbor contains large coverage gaps and some of the locations have mismatching bases for all reads. Combined these figures indicate that Clinical PathoScope has correctly identified the correct species in these clinical samples. (PDF 2 MB)
