# Supplementary figures and images for: Clinical PathoScope: rapid alignment and filtration for accurate pathogen identification in clinical samples using unassembled sequencing data
Source: BMC Bioinformatics. 2014 Aug 4;15(1):262. doi: 10.1186/1471-2105-15-262 (PMC4131054; doi:10.1186/1471-2105-15-262)

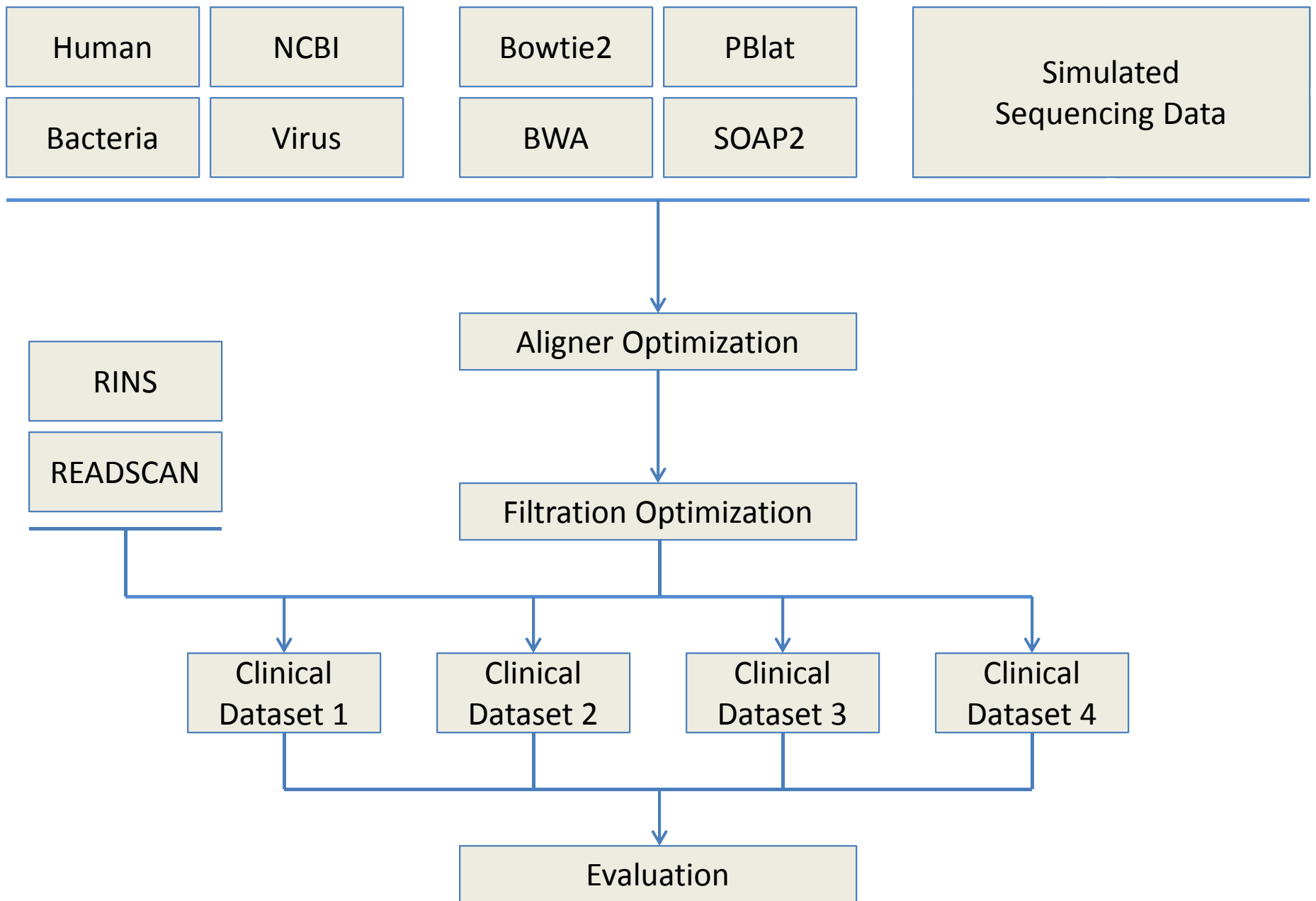

Supplement: Supplementary file 1 — Additional file 1: Workflow employed to develop the Clinical PathoScope pipeline. Three reference genome libraries were downloaded from NCBI. Four alignment algorithms were tested and evaluated on five simulated clinical sequencing samples. Each aligner was parameter tuned and optimized and Bowtie2 was selected as the choice aligner for the Clinical PathoScope pipeline. The order with which reads are aligned to the reference libraries was determined and the performance of Clinical PathoScope was evaluated using four clinical datasets. Furthermore, we compared our results against those produced by existing technologies. (PDF 19 KB) [file 12859_2013_6527_MOESM1_ESM.pdf]

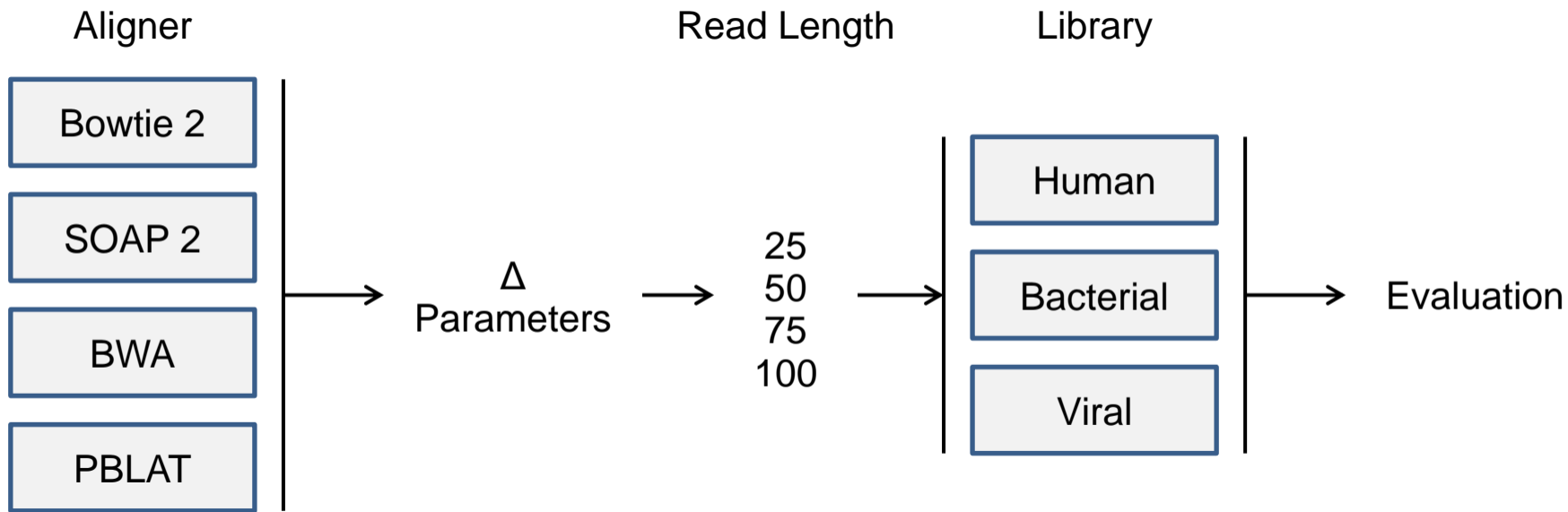

Supplement: Supplementary file 4 — Additional file 4: Alignment optimization variables and methods. The internal parameters for each of the four aligners were varied and tuned. Additionally, the length of each read aligned was varied. For each unique aligner-parameter-read length configuration, the sensitivity, specificity, and run time when aligning the simulated samples against the reference genome libraries was calculated. (PDF 76 KB) [file 12859_2013_6527_MOESM4_ESM.pdf]

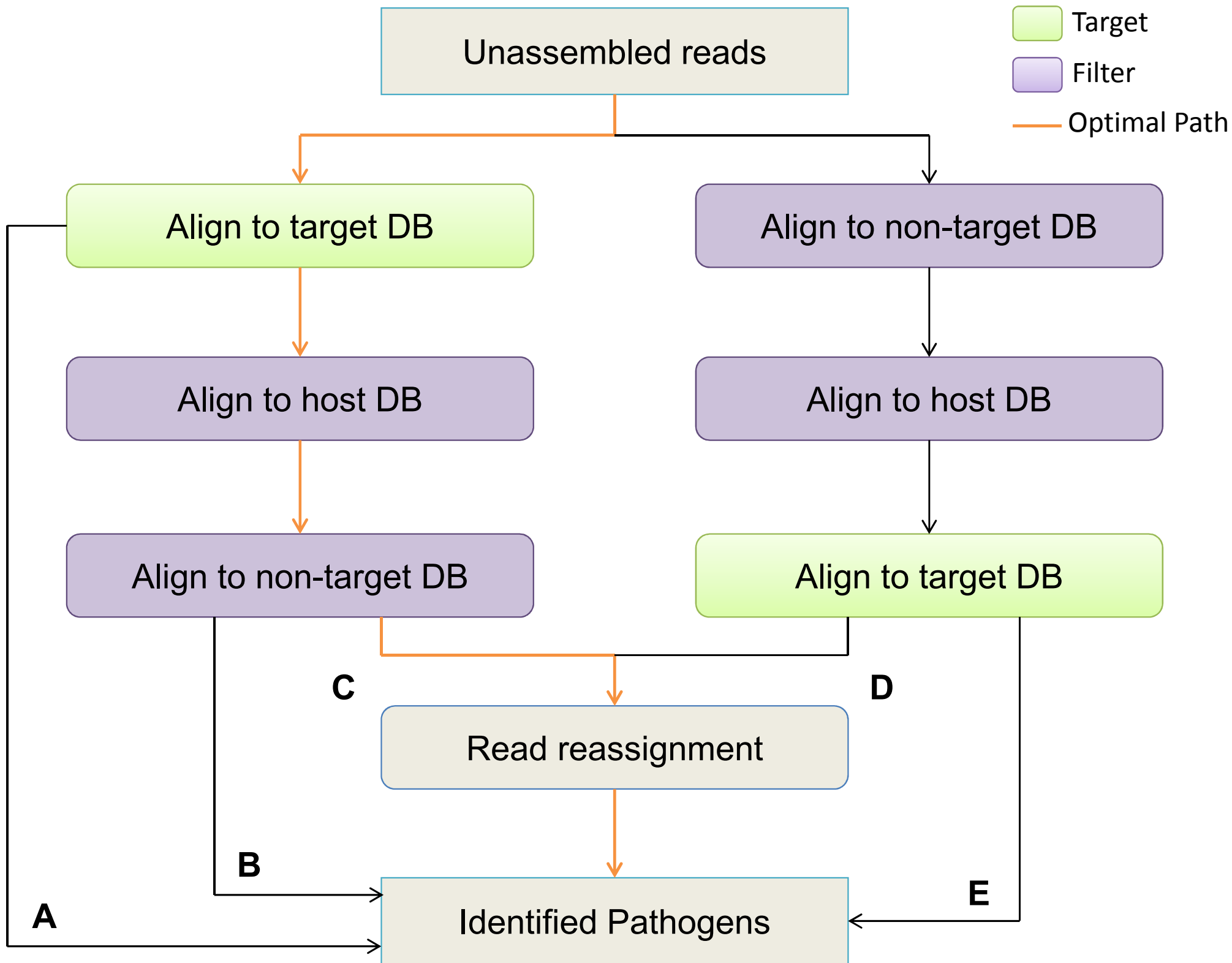

Supplement: Supplementary file 7 — Additional file 7: Subtraction and filtration optimization methods. Various filtration methods were tested in an effort to minimize computational burden and maximize accuracy. Approaches tested include A) Naïve Approach, B) Target Centric, C) Target Centric + Reassignment, D) Host Centric + Reassignment, and E) Host Centric. Post filtration, all reads are aligned against the target genome library. The resulting read alignments are reassigned to the correct genome of origin using the PathoScope Expectation Maximization algorithm. (PDF 39 KB) [file 12859_2013_6527_MOESM7_ESM.pdf]

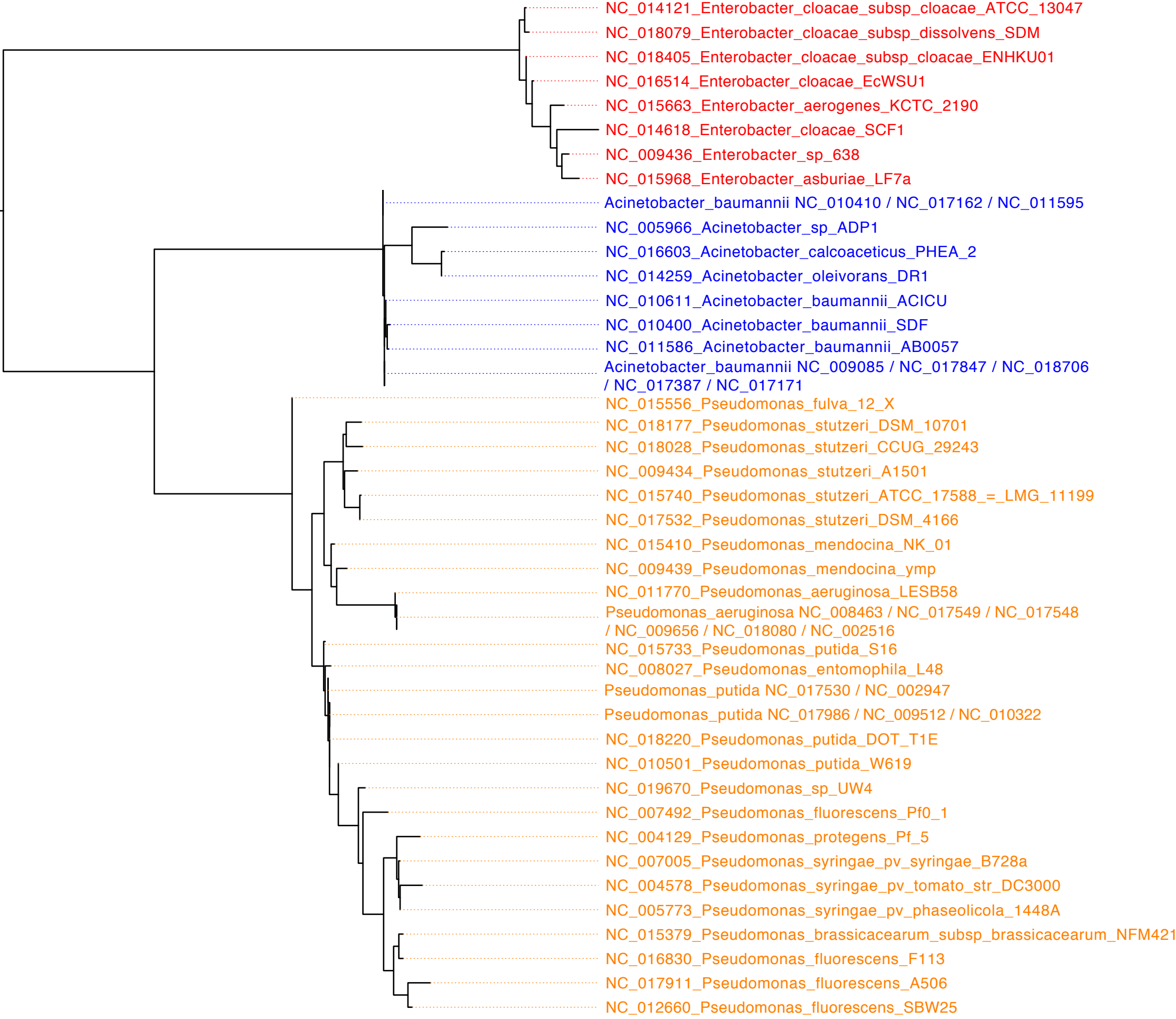

0.04

Supplement: Supplementary file 10 — Additional file 10: Phylogeny of 16S genes for genera found in clinical samples. We constructed a phylogenetic tree of 16S genes from all species in the reference library from the genera identified in the patient samples from the clinic. This tree was used to identify the nearest 16 s neighbor of the Clinical PathoScope diagnosis, and to check initial mapping read coverage of 16 s genes. (PDF 177 KB) [file 12859_2013_6527_MOESM10_ESM.pdf]
