# Supplementary material for: Clinical PathoScope: rapid alignment and filtration for accurate pathogen identification in clinical samples using unassembled sequencing data
Source: BMC Bioinformatics. 2014 Aug 4;15(1):262. doi: 10.1186/1471-2105-15-262 (PMC4131054; doi:10.1186/1471-2105-15-262)
Supplement: Supplementary file 5 — Additional file 5: Commands and versions of alignment algorithms evaluated. (DOCX 23 KB) [file 12859_2013_6527_MOESM5_ESM.docx]

Details of Aligner Comparison

The alignment algorithms (Bowtie 2, BWA , PBLAT , SOAP2) were evaluated and compared based on run time, sensitivity and specificity by aligning the simulated samples against the human, bacterial, and viral reference databases. Shown below for each aligner are all the necessary commands used to create the index and run the alignment. Also listed are the parameters and read lengths that were tested. To accommodate innate differences in human, bacterial, and viral sequences, different combinations of parameters were used for the different databases. At the end is a table summarizing the optimal parameters for each of the aligners for each of the databases.

1. Bowtie2

Version: 2.0.0

Create Index:

bowtie2-build –f database.fasta index

Run Alignment:

bowtie2 –phred33 –x index –U reads.fastq –S out.sam –p 8

Additional parameters tested for each database:

Human

For read lengths 25, 50*, 75, and 100:

--fast Same as: -D 5 -R 1 -N 0 -L 22 -i S,0,2.50)

--very-fast Same as: -D 10 -R 2 -N 0 -L 22 -i S,0,2.50

--sensitive Same as: -D 15 -R 2 -L 22 -i S,1,1.15 (default)

--very-sensitive* Same as: -D 20 -R 3 -N 0 -L 20 -i S,1,0.50

Bacteria and Virus

For read lengths 50 and 100*:

--fast Same as: -D 5 -R 1 -N 0 -L 22 -i S,0,2.50)

--very-fast Same as: -D 10 -R 2 -N 0 -L 22 -i S,0,2.50

--sensitive Same as: -D 15 -R 2 -L 22 -i S,1,1.15 (default)

--very-sensitive* Same as: -D 20 -R 3 -N 0 -L 20 -i S,1,0.50

*Produced best results

2. BWA

Version: 0.6.2

Create Index:

For small database (< 2GB):

bwa index –p index –a is small_database.fna

For large database:

bwa index –p index –a bwtsw large_database.fna

Run Alignment:

bwa aln index reads.fastq –t 8 –n x > out.sai

bwa samse index out.sai reads.fastq > out.sam

Parameter adjusted: -n x

Values tried for each database:

Human

For read length 25:

x = 0.04 (default), 1, 2, and 5

For read lengths 50*, 75, 100:

x = 0.04 (default), 2, 5, and 10*

Bacteria

For read lengths 50, 100:

x = 0.04 (default), 2, 5, and 10

Virus

For read lengths 50, 100*:

x = 0.04 (default), 2, 5, and 10*

*Produced best results

3. SOAP2

Version: 0.6.2

Create Index:

2bwt-builder database.fasta

Run Alignment:

soap –a reads.fastq –D index –o out.soap –unmapped.fastq –p 8 –v x

soap2sam.pl out.soap > out.sam

Parameter adjusted: -v x

Values tried for each database:

Human

For read length 25:

x = 1, 2 (default), and 5

For read lengths 50*, 75, 100:

x = 2 (default)*, 5, and 10

Bacteria

For read lengths 50* and 100:

x = 2 (default), 5, and 10*

Virus

For read lengths 50* and 100:

x = 2 (default), 5, and 10*

*Produced best results

4. PBLAT

Version: 2.0.0

Create Index:

faToTwoBit database.fasta index.2bit

Create file of overrepresented 11-mers

blat index.2bit /dev/null /dev/null -tileSize=11 -makeOoc=11.ooc -repMatch=300

Run Alignment:

cd directory_of_output/

blat index.2bit directory_of_output/reads.fasta –out=psl –threads=8

Parameter combinations tested

Human

For read lengths 25, 50*, 75 and 100:

-fastMap –ooc=11.ooc

–ooc=11.ooc

-minIdentity=80 –ooc=11.ooc*

-minIdentity=95 –ooc=11.ooc

Default (no –ooc=11.ooc)

Bacteria

For read lengths 50 and 100*:

-fastMap –ooc=11.ooc

–ooc=11.ooc*

-minIdentity=80 –ooc=11.ooc

-minIdentity=95 –ooc=11.ooc

Default (no –ooc=11.ooc)

Virus (no 11.ooc file was created)

For read lengths 50 and 100*:

Default*

-fastMap

-minIdentity=80

-minIdentity=95

*Produced best results

5. Summary table of parameters producing best results

|  | Human | | Virus | | Bacteria | |
| --- | --- | --- | --- | --- | --- | --- |
|  | Length | Param. | Length | Param | Length | Param |
| Bowtie2 | 50 | --very-sen | 100 | --very-sen | 100 | --very-sen |
| BWA | 50 | -n 10 | 50 | -n 10 | - | - |
| SOAP2 | 50 | -v 2 | 50 | -v 5 | 50 | -v 10 |
| PBLAT | 50 | minId= 80  -ooc=11.ooc | 100 | default | 100 | -ooc=11.ooc |
