# Supplementary material for: Clinical PathoScope: rapid alignment and filtration for accurate pathogen identification in clinical samples using unassembled sequencing data
Source: BMC Bioinformatics. 2014 Aug 4;15(1):262. doi: 10.1186/1471-2105-15-262 (PMC4131054; doi:10.1186/1471-2105-15-262)
Supplement: Supplementary file 11 — Additional file 11: Read coverage for 16S genes and nearest phylogenetic neighbors. A) F1, B) G1, and C) H1 16S clinical samples (top frame: overall coverage, bottom frame: ‘pileup’ plot for a selected sets of the reads). Coverage for the ‘nearest’ phylogenetic neighbor contains large coverage gaps and some of the locations have mismatching bases for all reads. Combined these figures indicate that Clinical PathoScope has correctly identified the correct species in these clinical samples. (PDF 2 MB) [file 12859_2013_6527_MOESM11_ESM.pdf]

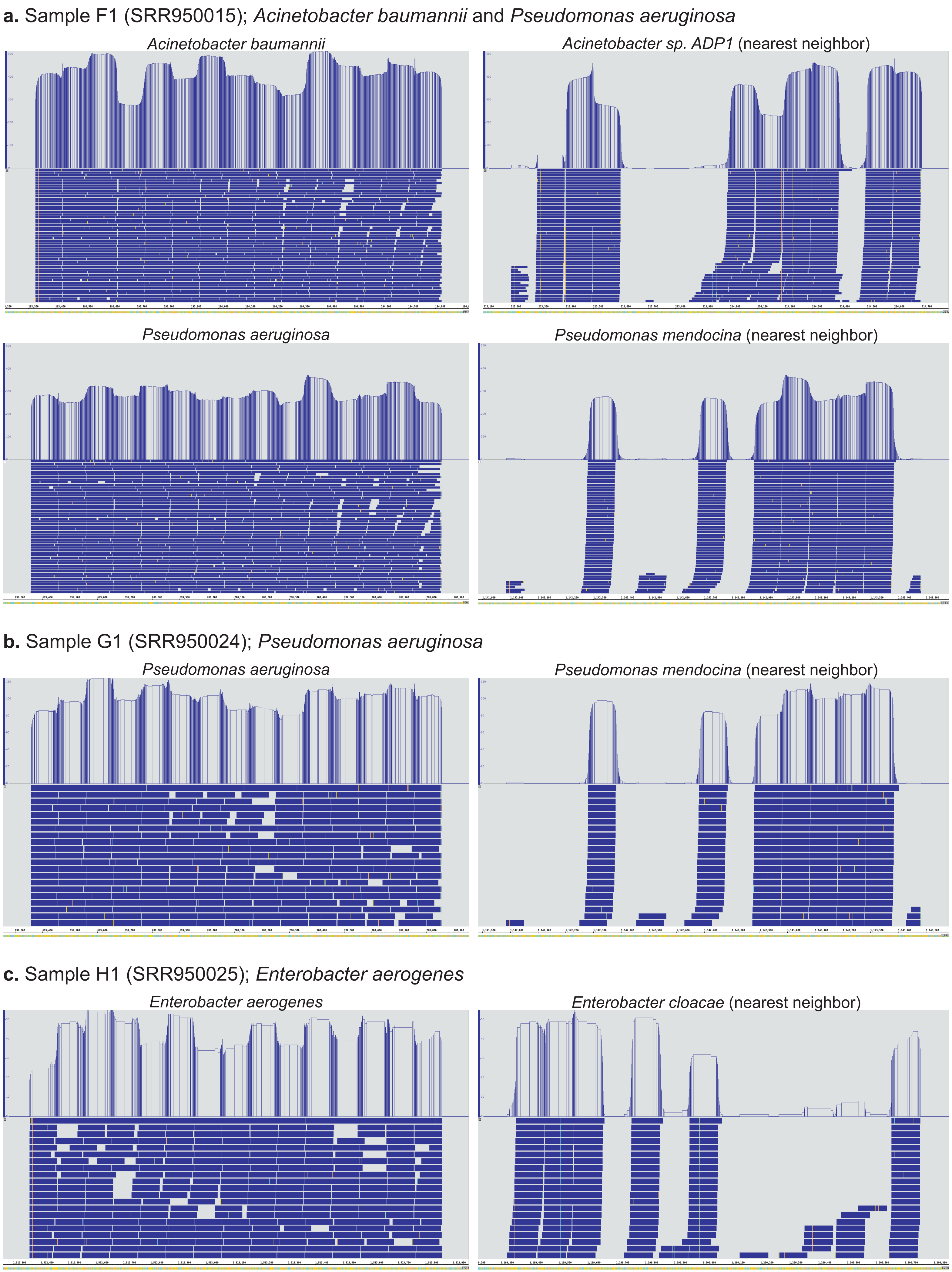
a. Sample F1 (SRR950015); Acinetobacter baumannii and Pseudomonas aeruginosa
Acinetobacter baumannii
Acinetobacter sp. ADP1 (nearest neighbor)
Pseudomonas aeruginosa
Pseudomonas mendocina (nearest neighbor)
b. Sample G1 (SRR950024); Pseudomonas aeruginosa
Pseudomonas aeruginosa
Pseudomonas mendocina (nearest neighbor)
c. Sample H1 (SRR950025); Enterobacter aerogenes
Enterobacter aerogenes
Enterobacter cloacae (nearest neighbor)
